# Impact of Donor−Recipient BMI Ratio on Survival Outcomes of Heart Transplant Recipients: A Retrospective Analysis Study

**DOI:** 10.1002/clc.70010

**Published:** 2024-09-04

**Authors:** Yucheng Zhong, Changdong Zhang, Yixuan Wang, Mei Liu, Xiaoke Shang, Nianguo Dong

**Affiliations:** ^1^ Department of Cardiovascular Surgery, Union Hospital, Tongji Medical College Huazhong University of Science and Technology Wuhan Hubei China; ^2^ Cardiac Laboratory, Department of Cardiovascular Surgery, Union Hospital, Tongji Medical College Huazhong University of Science and Technology Wuhan Hubei China

**Keywords:** BMI ratio, heart transplantation, risk of death, survival rate

## Abstract

**Objective:**

This study aimed to investigate the impact of the donor−recipient BMI ratio on the survival outcomes of heart transplant recipients.

**Methods:**

A retrospective analysis was conducted on 641 heart transplant patients who underwent surgery between September 2008 and June 2021. The BMI ratio (donor BMI divided by recipient BMI) was calculated for each patient. Kaplan−Meier survival analysis and Cox proportional hazards regression were performed to evaluate survival rates and determine the hazard ratio (HR) for mortality.

**Results:**

Significant differences were found in donor age and donor−recipient height ratio between the BMI ratio groups. The BMI ratio ≥ 1 group had a higher mean donor age (37.27 ± 10.54 years) compared to the BMI ratio < 1 group (34.72 ± 11.82 years, *p* = 0.008), and a slightly higher mean donor−recipient height ratio (1.02 ± 0.06 vs. 1.00 ± 0.05, *p* = 0.002). The Kaplan−Meier survival analysis indicated that the survival rate in the BMI ratio ≥ 1 group was significantly lower than in the BMI ratio < 1 group. Cox multivariate analysis, adjusted for confounding factors, revealed a HR of 1.50 (95% CI: 1.08−2.09) for mortality in patients with a BMI ratio ≥ 1. No significant differences were observed in ICU stay, postoperative hospitalization days, or total mechanical ventilation time between the groups.

**Conclusion:**

A higher donor−recipient BMI ratio was associated with an increased risk of mortality in heart transplant recipients.

## Graphical abstract

The study found that a higher donor−recipient BMI ratio is associated with an increased risk of mortality in heart transplant recipients. Kaplan−Meier survival analysis and Cox multivariate analysis confirmed that patients with a BMI ratio ≥ 1 had significantly lower survival rates compared to those with a BMI ratio < 1.

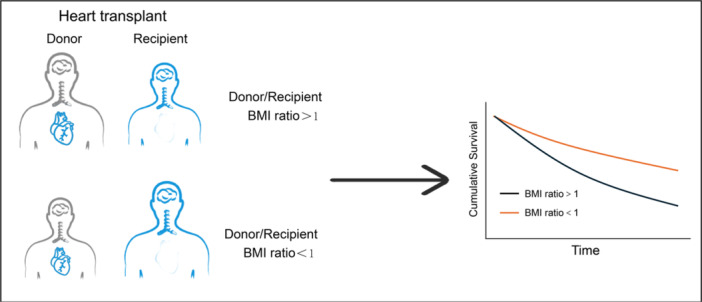

## 1 Introduction

There have been studies indicating that the life expectancy of patients with end‐stage heart failure is typically between 6 months and 1 year [1, 2, 3]. While pharmaceutical treatments may not be very effective for most of these patients, heart transplantation is considered the primary treatment option for end‐stage heart failure [4]. The International Society for Heart and Lung Transplantation (ISHLT) released heart transplant guidelines in 2006, recommending that patients should have a body mass index (BMI) of less than 30 kg/m^2^ or an ideal body weight percentage (expressed as a percentage of the average ideal weight for a given height and gender) of less than 140% before transplantation [5]. These guidelines state that a BMI greater than 30 kg/m^2^ before heart transplantation can result in unfavorable outcomes after the procedure [6]. Multiple studies have also indicated that higher BMI in patients increases the risks associated with heart transplantation. Patients with a BMI greater than 27.8 kg/m^2^ have shown significant improvement in survival after heart transplantation compared to underweight or normal‐weight patients [7]. Several other studies have also reached similar conclusions [8, 9, 10, 11].

Nevertheless, the relationship between BMI and posttransplant outcomes remains a subject of debate. While a study indicates a higher BMI is associated with improved survival after heart transplantation [12]. A study conducted by Russo et al. did not find a significant association between a BMI of 30 and 34.99 and higher incidence or mortality rates [13]. Clark et al. even found that patients with higher BMI had higher survival rates after heart transplantation [14]. However, these patients are also more prone to issues such as transplant rejection, coronary artery disease, and diabetes [15]. Further research is needed to better understand the complexities of this relationship.

In addition to the recipient, achieving an optimal size match between the donor and recipient is a significant challenge in heart transplantation, crucial for maximizing organ utilization and recipient survival. The 2010 ISHLT guidelines recommended size matching based on body weight, suggesting that the donor−recipient mismatch should generally not exceed 30%, and the difference between female donors and male recipients should not exceed 20% [16]. In other hand, body weight has been proven to be an imperfect predictor of heart size, and there are limitations in donor−recipient size matching in heart transplantation [17]. Predicted heart mass (PHM) is currently the most reliable indicator for donor−recipient size matching in heart transplantation. A donor−recipient size mismatch exceeding 20% in PHM independently predicts decreased survival rates. However, in obese recipients, the use of undersized hearts based on PHM does not correlate with decreased survival rates, and ideal recipient BMI matching is the sole size matching indicator predicting posttransplant mortality [18]. This suggests that ensuring BMI matching between the recipient and the donor is an important factor in reducing posttransplant mortality risk during organ transplantation. However, there is currently a lack of research investigating the relationship between donor−recipient BMI matching and post‐heart transplantation risks.

We aim to investigate whether the donor−recipient cardiac matching, as determined by the donor BMI/recipient BMI ratio, impacts the risks for post‐heart transplantation patients. One hypothesis of our study is that the donor−recipient BMI ratio may influence the risks associated with heart transplantation, particularly concerning survival rates, complication rates, and postoperative outcomes. Our findings could provide preliminary data and new research directions for future large‐scale validation studies to further confirm these hypotheses and gain a deeper understanding of their underlying mechanisms.

## 2 Materials and Methods

### 2.1 Clinic Data

Between September 2008 and June 2021, a total of 864 heart transplant surgeries were performed at our institution, involving modified donor hearts with hypothermic preservation. However, after excluding patients who were under 18 years of age or had missing age data (*n* = 119), patients with missing BMI data for donors or recipients (*n* = 83), and patients with missing donor gender data (*n* = 21), a final sample of 641 patients was included in the analysis (refer to Figure S1). Their follow‐up was completed ranging from 1 to 14 years, with a median follow‐up time of 6 years.

### 2.2 Clinical Data Collection

Clinical data for heart transplant recipients were collected from patient records, including demographic information, preoperative clinical assessments, intraoperative details, and postoperative outcomes. Variables included donor and recipient age, gender, BMI, donor−recipient height ratio, cause of donor death, and postoperative complications. This study was approved by the Ethics Committee of Tongji Medical College of Huazhong University of Science and Technology (No. IORG0003571).

### 2.3 Cardiac Donor Heart Preservation Method

Once brain death is confirmed in the donor, the surgical procedure commences by ensuring a sterile environment. A midline incision is made in the sternum, allowing access to the heart upon splitting it. The pericardium is then opened, and an occlusion clamp is applied to the ascending aorta. For non‐arrested donor hearts, the root of the aorta is perfused with 1000 mL of modified St. Thomas solution at 4°C. In the case of arrested donor hearts, 1000 mL of HTK solution at 8°C is used instead. Concurrently, quick incisions are made in the left and right atria to reduce the heart's volume and pressure. Subsequently, the pulmonary veins, superior and inferior vena cava, pulmonary artery, and aorta are sequentially severed while maintaining a perfusion pressure of 50−70 mmHg. Ice chips are strategically placed on the heart's surface to expedite the cooling process. Once removed, the donor heart is meticulously placed inside a triple‐layer sterile plastic bag and perfused through the aortic root with 1000−2000 mL of HTK solution at 8°C, completing the perfusion within a timeframe of 8−12 min. To ensure preservation and facilitate transportation, the donor heart is immersed in HTK solution containing histidine−tryptophan−ketoglutarate at a low temperature. Additionally, during heart preparation in the operating room, an extra 1000 mL of HTK solution is perfused through the aorta. The surgery is carried out utilizing the conventional double atrial or double caval venous technique under moderate hypothermia at 28°C.

### 2.4 Medical Treatment for Recipient

Basiliximab (20 mg) was administered intraoperatively and on the fourth day postoperation by intravenous pump for induction immunotherapy. This mediation was followed by a standard triple‐drug immunosuppression regimen, including cyclosporine A (CsA)/tacrolimus, mycophenolate mofetil, and prednisone. Prophylactic antibiotic therapy was discontinued in patients who exhibited no sign of infection 7 days after transplantation. Patients with elevated pulmonary pressure after operation were prescribed iloprost by inhaler and a 3‐month course of oral sildenafil [19]. Followed by endomyocardial biopsy, acute cellular rejection exceeding grade 2R according to the ISHLT criteria [20] was treated by administering 500 mg of methylprednisolone for 3 days and increasing the doses of immunosuppressive drugs.

### 2.5 Statistics

Statistical analysis was conducted using GraphPad Prism 9.0 software, and all tests were two‐sided. Survival rates were estimated using the Kaplan−Meier method, with survival curves plotted for different BMI ratio groups. The log‐rank test was used to compare survival distributions between these groups. The Cox multivariate analysis model was employed to account for potential confounding factors that might influence the mortality outcome. The model included adjustment for several variables, such as diagnostic category, recipient gender, postoperative IABP, postoperative ECMO, postoperative CRRT, donor age, donor−recipient gender, extracorporeal circulation time, aortic cross‐clamp time, preoperative cardiac ultrasound EF (M‐mode), and donor−recipient height ratio. A *p*‐value less than 0.05 was considered statistically significant.

## 3 Results

We compared baseline characteristics between the two groups based on BMI ratio. There were significant differences in donor age and donor−recipient height ratio. The BMI ratio ≥ 1 group had a higher mean donor age compared to the BMI ratio < 1 group (37.27 ± 10.54 vs. 34.72 ± 11.82 years, *p* = 0.008). Additionally, the BMI ratio ≥ 1 group had a slightly higher mean donor−recipient height ratio compared to the BMI ratio < 1 group (1.02 ± 0.06 vs. 1.00 ± 0.05, *p* = 0.002). No significant differences were observed in recipient age, donor heart preservation time, extracorporeal circulation time, aortic cross‐clamp time, and preoperative cardiac color EF (M‐mode) (Table 1). Table 2 compares baseline characteristics of count data between two groups. Significant differences were found in receptor sex (*p* = 0.003), diagnostic classification (*p* = 0.034), and postoperative IABP (*p* = 0.024). No significant differences were observed in donor cause of death classification, donor gender, postoperative ECMO, and postoperative CRRT.

**Table 1:** Comparison of baseline characteristics of quantitative data in the two groups.

Variables	BMI ratio group	Sample size (number of missing)	Mean ± SD	Statistic (*T*)	*p*
Recipient age	≤ 1	293 (0)	46.57 ± 13.55	−1.03	0.303
	< 1	348 (0)	48.26 ± 11.58		
	Difference		−1.69		
Donor heart preservation time (min)	≤ 1	293 (0)	325.71 ± 107.99	0.102	0.919
	< 1	348 (0)	320.17 ± 118.36		
	Difference		5.54		
Donor age (years)	≤ 1	293 (0)	37.27 ± 10.54	2.668	0.008
	< 1	348 (0)	34.72 ± 11.82		
	Difference		2.55		
Donor−recipient height ratio	≤ 1	293 (0)	1.02 ± 0.06	3.065	0.002
	< 1	348 (0)	1.00 ± 0.05		
	Difference		0.01		
Extracorporeal circulation time (min)	≤ 1	292 (1)	121.77 ± 69.83	0.213	0.831
	< 1	346 (2)	116.02 ± 37.50		
	Difference		5.76		
Aortic cross‐clamp time (min)	≤ 1	293 (0)	34.02 ± 17.09	−0.526	0.599
	< 1	347 (1)	32.86 ± 11.93		
	Difference		1.16		
Preoperative cardiac color EF (M‐mode)	≤ 1	289 (4)	28.01 ± 11.93	−0.687	0.492
	< 1	344 (4)	28.39 ± 11.89		
	Difference		−0.39		

Abbreviation: BMI, body mass index.

**Table 2:** Comparison of baseline characteristics of count data in two groups.

Variables	Level	BMI ratio ≥ 1	BMI ratio < 1	Statistic (*χ*)	*p*
Receptor sex	Male	214 (73.04)	288 (82.76)	8.851	0.003
	Female	79 (26.96)	60 (17.24)		
Diagnostic classification	CAD	53 (18.09)	84 (24.14)	8.650	0.034
	CM	179 (61.09)	218 (62.64)		
	Others	23 (7.85)	20 (5.75)		
	VHD	38 (12.97)	26 (7.47)		
Donor cause of death classification	Traumatic brain injury	161 (54.95)	204 (58.62)	4.162	0.125
	Cerebrovascular disease	101 (34.47)	96 (27.59)		
	Other	31 (10.58)	48 (13.79)		
Donor gender	Male	256 (87.37)	306 (87.93)	0.046	0.830
	Female	37 (12.63)	42 (12.07)		
Postoperative IABP	Yes	91 (31.06)	138 (39.66)	5.120	0.024
	No	202 (68.94)	210 (60.34)		
Postoperative ECMO	Yes	20 (6.83)	21 (6.03)	0.166	0.683
	No	273 (93.17)	327 (93.97)		
Postoperative CRRT	Yes	39 (13.64)	51 (14.83)	0.180	0.671
	No	247 (86.36)	293 (85.17)		

Abbreviations: BMI, body mass index; CAD, coronary artery disease; CM, cardiomyopathy; ECMO, extracorporeal membrane oxygenation; IABP, intra‐aortic balloon pump.

**Figure 1:**
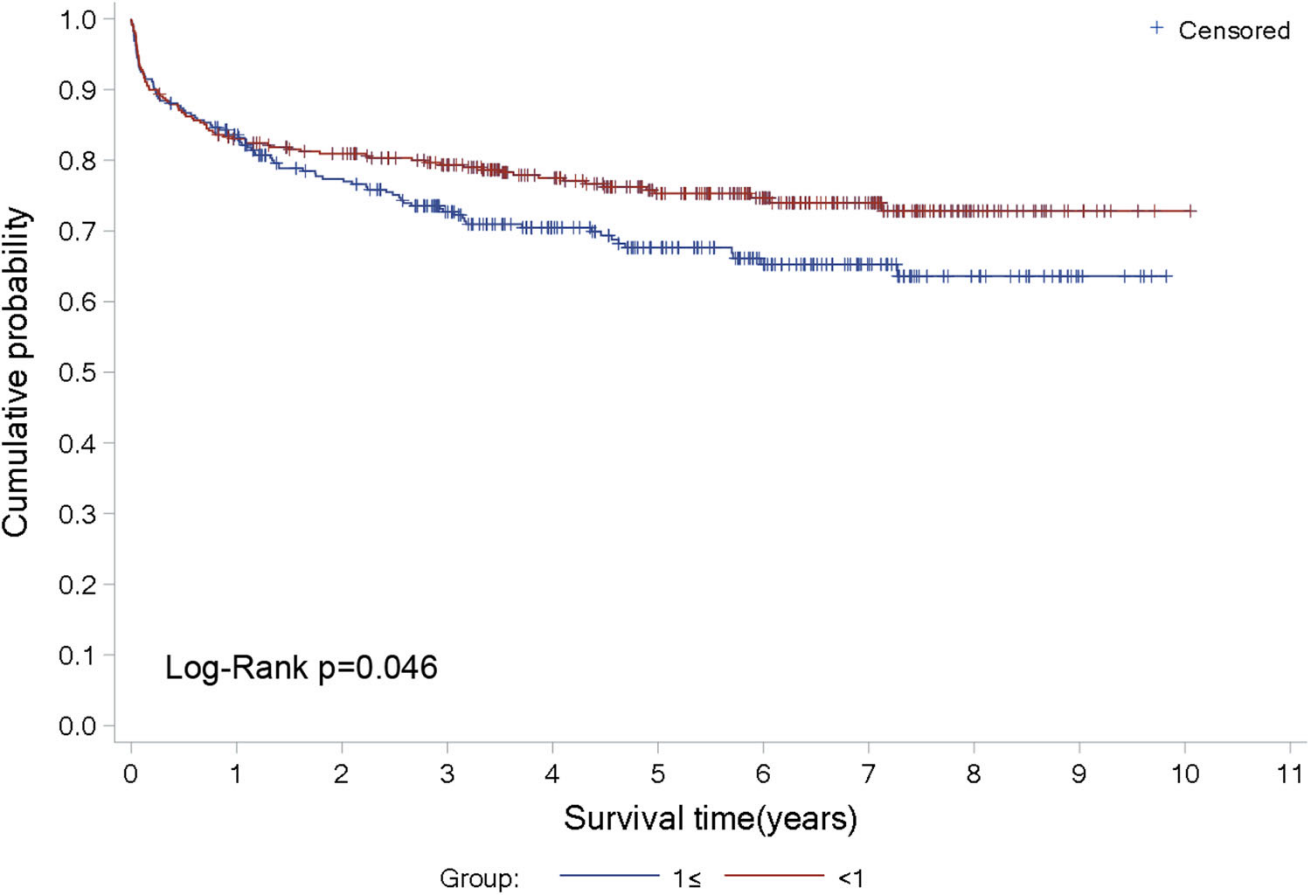
Survival curves for two BMI ratio groups. ≥ 1 indicates donor/acceptor BMI ratio greater than or equal to 1; < 1 indicates donor/acceptor BMI ratio < 1.

The log‐rank test showed that the survival rate in the BMI ratio ≥ 1 group was significantly lower than that in the BMI ratio < 1 group (*p* = 0.046) (Figure 1), suggesting that a higher donor−recipient BMI ratio is associated with poorer survival outcomes in heart transplant recipients.

The Cox multivariate analysis found that the HR for mortality in patients with BMI ratio ≥ 1, compared to those with BMI ratio < 1, was determined to be 1.50 (95% CI: 1.08−2.09) (Table 3).

**Table 3:** HRs for deaths occurring in patients with 1 ≤ BMI ratio versus BMI < 1.

Variables	Level	HR	95% CI	*p*
Diagnostic classification	CAD	2.058	1.002−4.228	0.0494
Diagnostic classification	CM	2.331	1.202−4.523	0.0123
Diagnostic classification	Others	4.037	1.754−9.292	0.0010
Sex of recipient	Male	0.724	0.360−1.459	0.3664
Postoperative IABP	Yes	1.336	0.929−1.922	0.1178
Postoperative ECMO	Yes	2.269	1.338−3.848	0.0024
Postoperative CRRT	Yes	6.296	4.356−9.099	< 0.0001
Donor gender	Male	0.974	0.523−1.812	0.9326
Recipient age	60 ≤ age	4.308	2.795−6.639	< 0.0001
Recipient age	50 ≤ age < 60	2.083	1.382−3.139	0.0005
Donor age	45 ≤ age	1.567	1.023−2.399	0.0389
Donor age	30 ≤ age < 40	1.025	0.676−1.555	0.9062
Extracorporeal circulation time (min)	< 90	0.790	0.434−1.440	0.4419
Extracorporeal circulation time (min)	90−130	0.939	0.622−1.417	0.7649
Aortic cross‐clamp time (min)	≤ 40	2.082	1.004−4.315	0.0486
Aortic cross‐clamp time (min)	25−40	1.642	0.889−3.031	0.1129
Preoperative cardiac ultrasound EF (M‐mode)	≤ 30	1.069	0.755−1.513	0.7087
Height ratio of donor and recipient	≤ 1.05	1.049	0.681−1.617	0.8273
BMI ratio	≤ 1	1.500	1.079−2.085	0.0159

Abbreviations: BMI, body mass index; CAD, coronary artery disease; CM, cardiomyopathy; CRRT, continuous renal replacement therapy; ECMO, extracorporeal membrane oxygenation; EF, ejection fraction; IABP, intra‐aortic balloon pump.

Table 4 presents the effect of BMI ratio on hospitalization and duration of mechanical ventilation. The results suggest that there were no significant differences in ICU stay, postoperative hospitalization days, and total mechanical ventilation time between the BMI ratio ≥ 1 and BMI ratio < 1 group.

**Table 4:** Effect of BMI ratio on hospitalization and duration of mechanical ventilation.

Variables	BMI ratio group	Sample size (number of missing)	Mean ± SD	Statistic (*T*)	*p*
ICU stay (h)	≤ 1	288 (5)	245.26 ± 218.28	−1.443	0.149
	< 1	342 (6)	267.00 ± 254.61		
	Difference		−21.74		
Postoperative hospitalization days (days)	≤ 1	288 (5)	37.31 ± 21.27	−1.523	0.128
	< 1	342 (6)	39.57 ± 20.51		
	Difference		−2.27		
Total mechanical ventilation time (min)	≤ 1	289 (4)	4384.66 ± 9336.57	−1.328	0.184
	< 1	346 (2)	4745.21 ± 11 682.59		
	Difference		−360.56		

## 4 Discussion

Obesity in recipients has been identified as a risk factor for post‐heart transplantation complications in some studies. For instance, obese patients have shown a significant correlation with increased mortality rates after transplantation [21]. BMI has been shown to have a significant association with the risk of heart failure. For every 1 unit increase in BMI, the incidence of heart failure increases by 5% in males and 7% in females [22]. A study by Khan et al. found that BMI has the strongest association with heart failure, with obese individuals having a fivefold increased risk of developing heart failure [23]. Healy et al. [24] found that BMI is an independent risk factor for mortality, with higher BMI associated with increased death rates. In their retrospective analysis, the median BMI among patients was 28, indicating that patients with a normal weight BMI of 20−25 had relatively lower posttransplant mortality risk. However, other studies have suggested that high recipient BMI is not a risk factor for post‐heart transplantation mortality. Russo et al. [13] using the UNOS database, found no significant association between obesity (BMI 30−34.96) and long‐term mortality in heart transplant recipients. Weiss et al. [25] in their assessment, concluded that patients with higher BMI levels did not experience increased short‐term mortality rates within 30 days, 90 days, or 1 year after heart transplantation.

The BMI ratio ≥ 1 or BMI ratio < 1 in our study results does not directly reflect the weight of the donor or recipient. In the case of BMI ratio ≥ 1, if the recipient has a high BMI indicating overweight, it may appear that the higher mortality risk can be explained by their high BMI. However, in situations where the BMI ratio ≥ 1, it could also be possible that the recipient's BMI falls within the normal range, and the donor's BMI is also within the normal range. In such cases, the higher mortality risk cannot be solely attributed to the recipient's high BMI. Similar considerations apply to the results of the BMI ratio < 1 group. Therefore, it is not appropriate to interpret the results solely based on the recipient's high or low BMI. Our study found that patients with a BMI ratio ≥ 1 had a higher risk of mortality compared to patients with a BMI ratio < 1. A BMI ratio ≥ 1 indicates that the donor's BMI is higher than the recipient's BMI. This means that, in comparison to the recipient, the donor heart comes from an individual who is relatively heavier. On the other hand, a BMI ratio < 1 signifies that the donor's BMI is lower than the recipient's BMI, indicating that the donor heart comes from an individual who is relatively lighter compared to the recipient. We speculate that one reason why patients with a BMI ratio ≥ 1 have a higher risk of mortality compared to those with a BMI ratio < 1 is that the group with a BMI ratio < 1 has better body size matching between the donor and recipient. Body size matching indicators include PHM matching based on the ideal recipient weight, ideal weight matching, and ideal BMI matching. Kransdorf et al. [26] evaluated the ability of five body size matching indicators (PHM, weight, height, BMI, and body surface area) to predict 1‐year mortality after heart transplantation and found that PHM was the best indicator for predicting posttransplant mortality in terms of donor−recipient size matching. Kim et al. [18] conducted a study and found that recipient BMI matching was the only body size matching indicator that could predict posttransplant mortality in obese patients, compared to recipient weight matching and PHM matching. Our findings suggest that the relative body size between the donor and recipient, as indicated by the BMI ratio, might influence posttransplant outcomes. However, it is important to note that the BMI ratio alone does not provide a complete understanding of the complexities involved in heart transplantation.

Our study has certain limitations. The most significant one is that we currently lack a clear explanation for the observed differences between the BMI ratio ≥ 1 and BMI ratio < 1 group. Simply attributing it to the donor being lighter than the recipient is insufficient. On the other hand, as this study is a retrospective observational analysis, we cannot fully eliminate the risk of bias and confounding factors. Although we attempted to adjust for known confounders in the multivariate analysis, which were selected based on existing literature and baseline differences between the two groups (BMI ratio ≥ 1 and BMI ratio < 1), unmeasured or residual confounders may still impact our findings. Additionally, the inherent limitations of a retrospective design prevent us from establishing a causal relationship between donor−recipient BMI ratio and posttransplant survival; we can only explore their association. Future studies should employ a prospective design and include a more diverse population to further validate our current findings and to better understand the impact of donor−recipient weight matching on heart transplant outcomes.

## Conflicts of Interest

The authors declare no conflicts of interest.

## Supporting information

Supplementary Figure 1. The flowchart of this study.

## Data Availability

The data sets generated and analyzed during the current study are available from the corresponding author on reasonable request.
