# Supplementary figures and images for: Impact of Donor−Recipient BMI Ratio on Survival Outcomes of Heart Transplant Recipients: A Retrospective Analysis Study
Source: Clin Cardiol. 2024 Sep 4;47(9):e70010. doi: 10.1002/clc.70010 (PMC11375284; doi:10.1002/clc.70010)

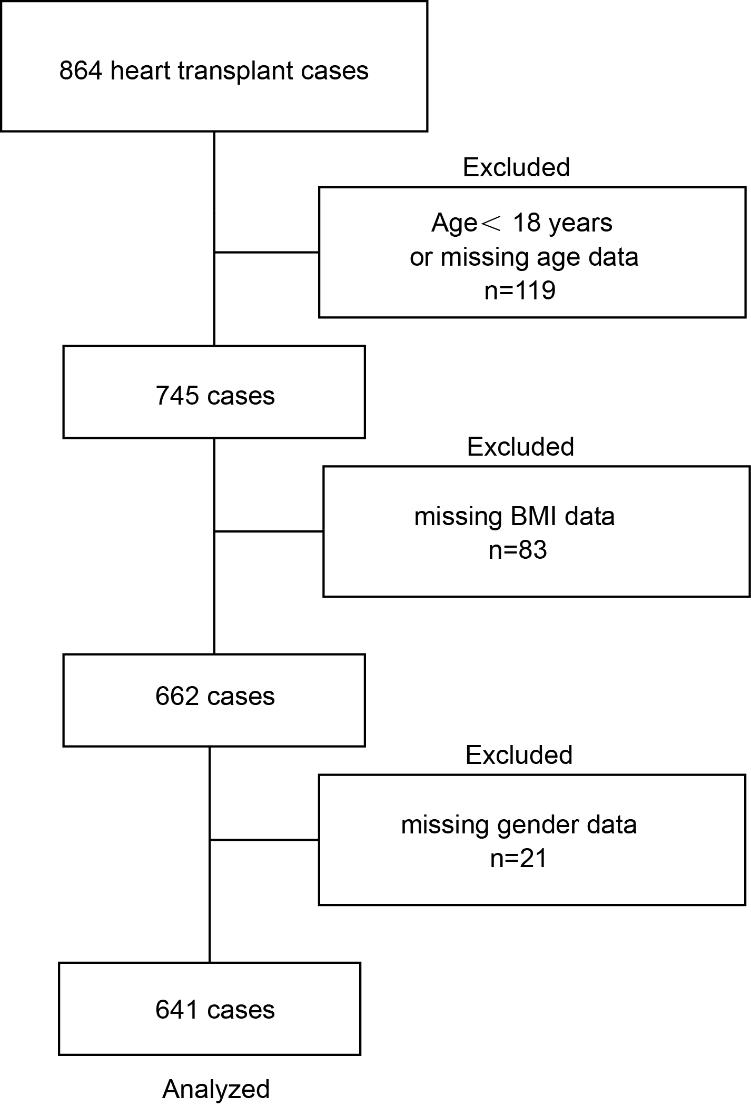

Supplement: Supplementary file 1 — Supplementary Figure 1. The flowchart of this study. [file CLC-47-e70010-s001.jpg]
